# Measurement training and feedback system for implementation of evidence-based treatment for adolescent externalizing problems: protocol for a randomized trial of pragmatic clinician training

**DOI:** 10.1186/s13063-019-3783-8

**Published:** 2019-12-10

**Authors:** Aaron Hogue, Molly Bobek, Alexandra MacLean, Nicole Porter, Amanda Jensen-Doss, Craig E. Henderson

**Affiliations:** 1 Center on Addiction, New York, NY USA; 2 Department of Psychology, University of Miami, Miami, FL USA; 3 Department of Psychology, Sam Houston State University, Huntsville, TX USA

**Keywords:** Adolescent conduct problems, Adolescent substance use, Family therapy, Cognitive behavioral therapy, Measurement feedback system, Training and consultation

## Abstract

**Background:**

Innovations in clinical training and support that enhance fidelity to evidence-based treatment (EBT) for adolescent behavior problems are sorely needed. This study will develop an online training system to address this gap: Measurement Training and Feedback System for Implementation (MTFS-I). Using procedures intended to be practical and sustainable, MTFS-I is designed to increase two aspects of therapist behavior that are fundamental to boosting EBT fidelity: therapist self-monitoring of EBT delivery, and therapist utilization of core techniques of EBTs in treatment sessions. This version of MTFS-I focuses on two empirically supported treatment approaches for adolescent conduct and substance use problems: family therapy and cognitive behavioral therapy (CBT).

**Methods/design:**

MTFS-I expands on conventional measurement feedback systems for client outcomes by adding training in observational coding to promote EBT self-monitoring and focusing on implementation of EBT treatment techniques. It has two primary components. (1) The *training component*, delivered weekly in two connected parts, involves self-monitored learning modules containing brief clinical descriptions of core EBT techniques and mock session coding exercises based on 5–8 min video segments that illustrate delivery of core techniques. (2) The *feedback component* summarizes aggregated therapist-reported data on EBT techniques used with their active caseloads. MTFS-I is hosted online and requires approximately 20 min per week to complete for each treatment approach. This randomized trial will first collect data on existing delivery of family therapy and CBT techniques for youth in outpatient behavioral health sites (Baseline phase). It will then randomize site clinicians to two study conditions (Implementation phase): Training Only versus Training + Feedback + Consultation. Therapists will choose whether to train in family therapy, CBT, or both. Study aims will compare clinician performance across study phase and between study conditions on MTFS-I uptake, reliability and accuracy in EBT self-monitoring, and utilization of EBT techniques in treatment sessions (based on observer coding of audiotapes).

**Discussion:**

Study contributions to implementation science and considerations of MTFS-I sustainability are discussed.

**Trial registration:**

ClinicalTrials.gov, NCT03722654. Registered on 29 October 2018.

## Background

### Family therapy and cognitive behavioral therapy are prime candidates for improving the quality of treatment for adolescent externalizing problems

Disseminating effective methods to improve the quality of available treatment services for adolescent externalizing problems (AEPs) in behavioral care is an urgent public health priority. There remains a troubling “quality gap” between behavioral treatments proven in controlled research versus those commonly practiced in usual care [1]. This gap is highly evident for AEPs, which encompass serious conduct problems, delinquency, and substance misuse. AEPs are the most common adolescent behavioral issues in specialty care, which follows from their high prevalence rates. In the USA, for example, conduct disorder has a 1-year population prevalence among youth ranging from 2 to 10% [2]; 15% of adolescents meet diagnostic criteria for alcohol use disorder and 16% for substance use disorder by age 18 [3]; and 31 million youth are involved in the juvenile justice system, with approximately 1.5 million new youth arrested each year [4]. Moreover, comorbidity between conduct and substance use problems is the rule rather than the exception among clinic-referred teenagers [5]. Yet standard treatment quality for AEPs is considered mediocre to inadequate due to a host of factors headlined by the absence or modest quality of evidence-based services, insufficient provider training, and little or lax quality monitoring [6, 7].

Two behavioral treatment approaches are prime candidates for upgrading the quality of AEP treatment services. Both family therapy (FT) and cognitive behavioral therapy (CBT) have excellent efficacy evidence for AEPs in both research and community settings [7, 8]. Each has strong support from research conducted in several countries for treating serious conduct problems [7–9], delinquency [7, 10], and substance use [6], and each has several manualized versions proven efficacious across the AEP range. Due to this extensive evidence base, there is incentive from clinical providers and payers to deliver these approaches in routine care: Both are now approved for treating AEPs by third-payer insurance plans and by regulatory agencies that govern licensed treatment providers (e.g.,). Notably, therapists report that both are highly valued in everyday practice [11, 12].

### Boosting fidelity to evidence-based treatment is a royal road to improving quality

An efficient pathway to improving the quality of behavioral health services is to increase the adoption and delivery of evidence-based treatments (EBTs) in usual care [1]. However, there is a caveat: For EBTs to be effective in front-line settings, they must be delivered with sufficient fidelity to the core principles and techniques of the approaches they represent. This remains a most difficult challenge for which innovative solutions are sorely needed [13]. With regard to AEPs, controlled studies have shown that strong fidelity to the FT and CBT approaches predicts improved client outcomes in both efficacy [14] and effectiveness [15] studies. Moreover, greater utilization of core EBT techniques for AEPs predicts better youth outcomes even when services are provided by community clinicians not trained in manualized treatments [16]. Yet at this time neither FT nor CBT is widely implemented with fidelity in community clinics that treat AEPs [1].

Given that stronger fidelity to EBTs can lead to improved outcomes for youth with AEPs, innovations in clinician training and support designed to increase fidelity to the FT and CBT approaches are sorely needed. The current study will develop a pragmatic online training system to achieve this goal. The study protocol is a randomized trial that will test a Measurement Training and Feedback System for Implementation (MTFS-I; see [17]) to increase fidelity to FT and CBT in behavioral care. MTFS-I is an example of a “learning” quality improvement system in which EBT delivery activities are carried out incrementally, implementation and sustainability data are regularly reviewed, and continuous EBT modifications are made to increase fit and/or feasibility ([18]). Learning systems are intended to ingrain data-driven decision-making into the procedural routines of agencies.

As next described, MTFS-I is designed to increase two aspects of therapist behavior that are fundamental to boosting EBT fidelity in a manner that is sustainable with typical agency resources [19]: therapist self-monitoring of EBT fidelity, and therapist utilization of EBT techniques in treatment sessions. MTFS-I traffics in EBT “core elements” [20] rather than full manualized protocols. EBT core elements are operationalized as discrete treatment techniques that are common ingredients of multiple EBT protocols for a given disorder. Core elements are considered easier to master than full EBT manuals, and they equip clinicians with a diverse portfolio of techniques that can be judiciously applied to clients presenting with comorbid, heterogeneous, and/or emerging clinical problems, making them well suited for the eclectic treatment practices that constitute usual care.

### EBT fidelity boost, part 1: train therapists to self-monitor by mimicking observational coding methods

One major step toward boosting the capacity of community therapists to implement EBTs with fidelity is improving their ability to accurately monitor (i.e., recognize and assess) the EBTs they are expected to deliver. There is consensus that training clinicians to self-report accurately on EBT delivery is a pragmatic strategy for tracking and ultimately improving EBT fidelity in usual care [19]. Self-report fidelity procedures are quick, inexpensive, non-intrusive, and compatible with electronic medical record systems, making them sustainable in everyday service contexts. They are also flexible in that they can capture fidelity to specific treatment manuals or to core treatment techniques associated with evidence-based approaches [21].

However, for fidelity self-monitoring to be useful, there remains a major psychometric hurdle to clear: Studies attempting to confirm the validity of therapist self-ratings of EBT fidelity by comparing them with observer ratings have mostly produced disappointing results, casting doubt on the accuracy with which therapists can judge their own performance. Research with both adult and youth populations has logged modest to weak concordance between therapist and observer reports of fidelity to various EBTs. And although a handful of studies (e.g., [21]) have found moderate *reliability* (i.e., adequate correlations) between therapists and observers when reporting on EBT utilization, these studies also found that therapists showed uniform *inaccuracy*: They reported much greater average use of EBTs (i.e., significantly higher mean fidelity scores) than did observers. Overall, weak concordance with trained observers is a universal therapist bias that affects research-trained clinicians delivering manualized EBTs as well as clinicians in usual care.

This study will develop an innovative strategy for improving therapist self-monitoring (reliability and accuracy) of EBT fidelity: teach clinicians to be fluent in self-rating by employing rigorous training procedures analogous to those used to train observational fidelity raters in controlled research. Unfortunately, gold-standard observational methods are resource-intensive, requiring numerous hours for introducing the coding scheme, reviewing recordings outside training sessions to calibrate scoring, and convening meetings throughout coding activities to prevent coder drift. Directly transporting these methods from research labs to everyday care—that is, training agency staff to reliably assess EBT use by supervisees or colleagues—is well beyond the resource capacity of most providers. However, some have asserted that by mimicking observational methods when training community therapists to self-monitor, it is possible to improve the reliability and accuracy of therapist-reported EBT fidelity [19]. Is this approach feasible? Front-line therapists can be trained to report EBT fidelity reliably under *ideal* conditions, that is, as research-trained judges in lab settings [14]. But can they be trained to self-monitor fidelity under the *pragmatic* conditions that prevail in usual care?

The self-monitored training method most likely to succeed is online instruction, a research-proven approach wherein training content can be presented in a user-tailored manner [22]. Brief online methods appear to be an excellent surrogate for gold-standard methods to increase therapist capacity to validly assess their own EBT fidelity. This study’s approach to mimicking observer training is as follows: Over the course of 1 year, clinicians will receive weekly email prompts to view online, brief (5–8 min) video vignettes, each demonstrating a selection of core FT and CBT treatment techniques they are hoping to deliver. After viewing each vignette, therapists submit fidelity rating scores for a selection of techniques (covering the full roster over time); they are then immediately shown gold-standard rating scores for the given vignette for direct comparison with their own ratings.

### EBT fidelity boost, part 2: adapt measurement feedback systems to strengthen EBT utilization

Another major step toward boosting EBT fidelity in usual care involves adapting measurement-based care (MBC) methods. MBC is a performance feedback loop in which a given quality metric is continuously monitored by a clinician to gauge case progress and support clinical decision-making [23]. To date MBC has been used in behavioral care primarily to monitor client outcomes, wherein the outcome metrics are standardized measures of client functioning. MBC feedback loops are often supported by the use of measurement feedback system (MFS) technology that generates easy-to-digest data reports providing summary appraisals of client progress compared to a desired benchmark. MBC has led to impressive gains in treatment outcomes across diverse adult clinical samples (e.g., [24, 25]). Also, clinicians trained in MBC can develop positive attitudes toward it [25]. MBC research with youth samples is new, but there is strong enthusiasm about reaping comparable benefits [25].

MBC success for client outcomes has generated enthusiasm for developing comparable procedures for routine feedback of treatment implementation data [23, 24]. When attuned to treatment delivery processes such as EBT fidelity, MBC can serve as a functional quality assurance procedure with broad dissemination potential for youth behavioral care [23]. This study’s approach to adapting MBC for implementation data involves summarizing therapist self-reports of FT and CBT technique delivery in user-friendly infographics distributed to clinicians and supervisors on a monthly basis. MFS technology has already been incorporated into quality procedures to bolster fidelity for several standardized protocols [23, 24], although the validity of therapist-reported EBT use for these protocol-based systems is not yet well established via concordance with observer ratings.

There is also reason to believe that adapted MBC methods providing feedback reports on EBT implementation can increase EBT use even if therapists are not ultimately accurate in EBT self-monitoring. Regular review of feedback reports by clinicians and supervisors can spur direct comparison between therapist self-reports of EBT fidelity versus agency-specified fidelity benchmarks, precipitating self-correction responses that should guide movement toward agency fidelity goals [24]. Also, case feedback along multiple dimensions, including routine feedback on treatment processes like EBT fidelity, is thought to optimize change potential [23]. And in addition to effects from EBT implementation feedback, EBT utilization can be increased by the online training procedures described above: Providing therapists with videos that model high-fidelity FT and CBT techniques creates a forum for observational learning of EBT delivery and prompts attention to EBT-infused service delivery.

### Protocol summary and specific aims

**Fig. 1:**
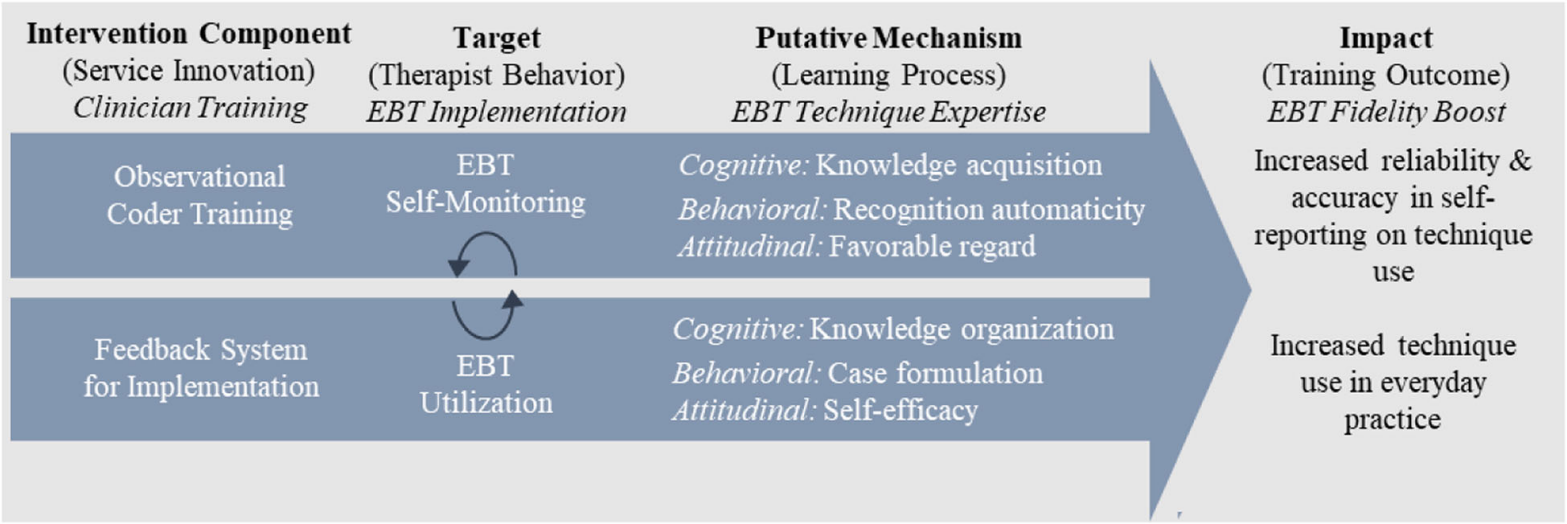
Training process schematic: Measurement Training and Feedback System for Implementation (MTFS-I) of FT and CBT techniques

Family therapy and CBT have strong effectiveness evidence for AEPs but remain widely underutilized in clinical practice. This study tests a clinician training system, MTFS-I, designed to increase delivery of these EBTs in behavioral health services internationally. MTFS-I expands on conventional MFS technology by (1) adding *Training* in observational coding to promote EBT self-monitoring and (2) focusing on *Implementation* in the form of fidelity to core EBT techniques. Figure 1 depicts the basic training process by which MTFS-I is meant to produce effects on EBT self-monitoring and technique use, including the intervention components, targets, putative mechanisms, and impact of the training. The observational coder training and implementation feedback components contained in MTFS-I are functionally symbiotic: Training is meant to ensure that clinicians generate valid self-report data to anchor feedback reports, and feedback reports supply motivational context for dedicated participation in self-monitored training. The putative learning mechanisms—cognitive, behavioral, attitudinal [26]—activated by the two intervention components await confirmation and articulation via direct testing. It is critical to note that MTFS-I is not intended to be a “replacement approach” in which clinicians are trained to implement new EBTs, but rather an “augmentation approach” aimed at enhancing expertise for EBTs already endorsed and practiced to some degree [13].

This randomized trial will first collect data on existing delivery of FT and CBT techniques for youth with AEPs in four outpatient behavioral health sites (Baseline phase). It will then experimentally compare the boost in FT and CBT fidelity produced when clinicians are randomized to one of two study conditions (Implementation phase): Training Only versus Training + Feedback + Consultation. Study data will include (1) post-session therapist-reported checklists on EBT use and (2) audiotapes of treatment sessions that will be observationally coded by research staff. Study Aim 1 will compare Baseline versus Implementation phases in EBT self-monitoring and EBT utilization, combining across sites. We expect that both self-monitoring (reliability, accuracy) and technique use (FT, CBT) will be significantly greater during the Implementation phase, after initiation of MTFS-I training. Study Aim 2 will experimentally test the effects of one MTFS-I component (Training Only) versus a full MTFS-I package that includes both system components plus ongoing expert facilitation (Training + Feedback + Consultation). We expect that Training + Feedback + Consultation will be superior to Training Only in promoting EBT self-monitoring and technique use. We will also examine the strength of correlation between self-monitoring and technique use via within-subject (comparing study phases) and between-subject (comparing study conditions) analyses.

## Methods/design

### Trial design

**Fig. 2:**
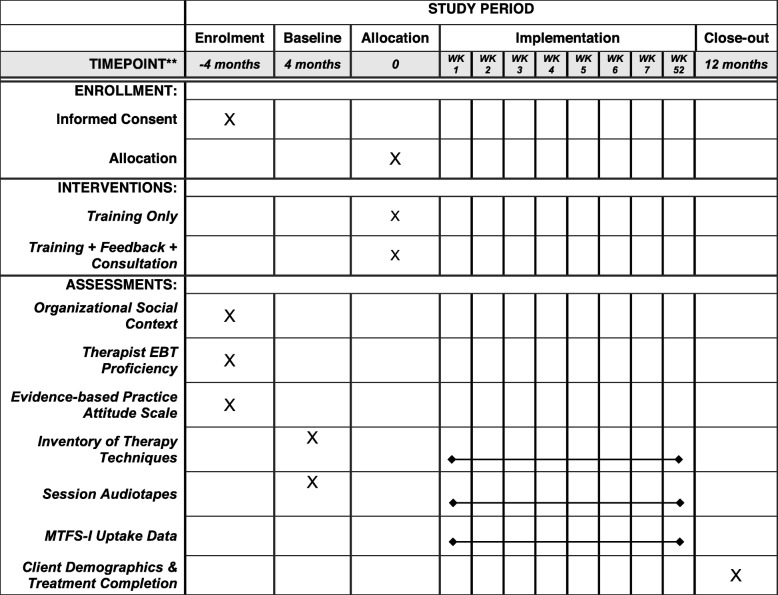
SPIRIT diagram depicting the schedule of trial enrollment, interventions, and assessments

The trial design is a two-group randomized trial with baseline comparison: Following a 4-month Baseline phase, therapists working in behavioral health treatment sites will be randomized across two experimental conditions, Training Only versus Training + Feedback + Consultation, for a 1-year Implementation phase. During both study phases we will collect EBT fidelity data (therapist-reported checklists, session audiotapes); during the Implementation phase we will also collect MTFS-I uptake data (training and consultation activity). In Aim 1, by comparing Baseline versus Implementation data, we can examine the effectiveness of the MTFS-I training component for enhancing EBT fidelity across study sites, yielding proof-of-concept data in accord with the well-established Stage Model of behavioral treatment development [27]. In Aim 2, by experimentally comparing Training Only versus Training + Feedback + Consultation, we can test the added value of MBC procedures combined with expert consultation procedures [26] for enhancing MTFS-I benefits. Also the quasi-experimental analysis of Baseline data (averaged across all study therapists) versus Training Only condition data will shed light on the unique effects of the online training component, which is the most pragmatic feature of the overall MTFS-I package. Comparing averaged Baseline-phase data versus the Training + Feedback + Consultation condition will also provide insight on the full MTFS-I effects. It was not possible to include a no-intervention or waitlist control group because sites unilaterally preferred that all study therapists receive a clinically meaningful training experience. To maximize clinical utility and trainee motivation, each study therapist will select whether they want to train in FT, CBT, or both; training in both approaches will double the time commitment (to approximately 40 total min per week) for the given trainee. Figure 2 is the Standard Protocol Items: Recommendations for Interventional Trials (SPIRIT) diagram depicting the schedule of trial enrollment, interventions, and assessments. Additional file 3 presents the SPIRIT checklist. Study sites and eligibility, sample size, randomization, and contamination procedures.

**Fig. 3:**
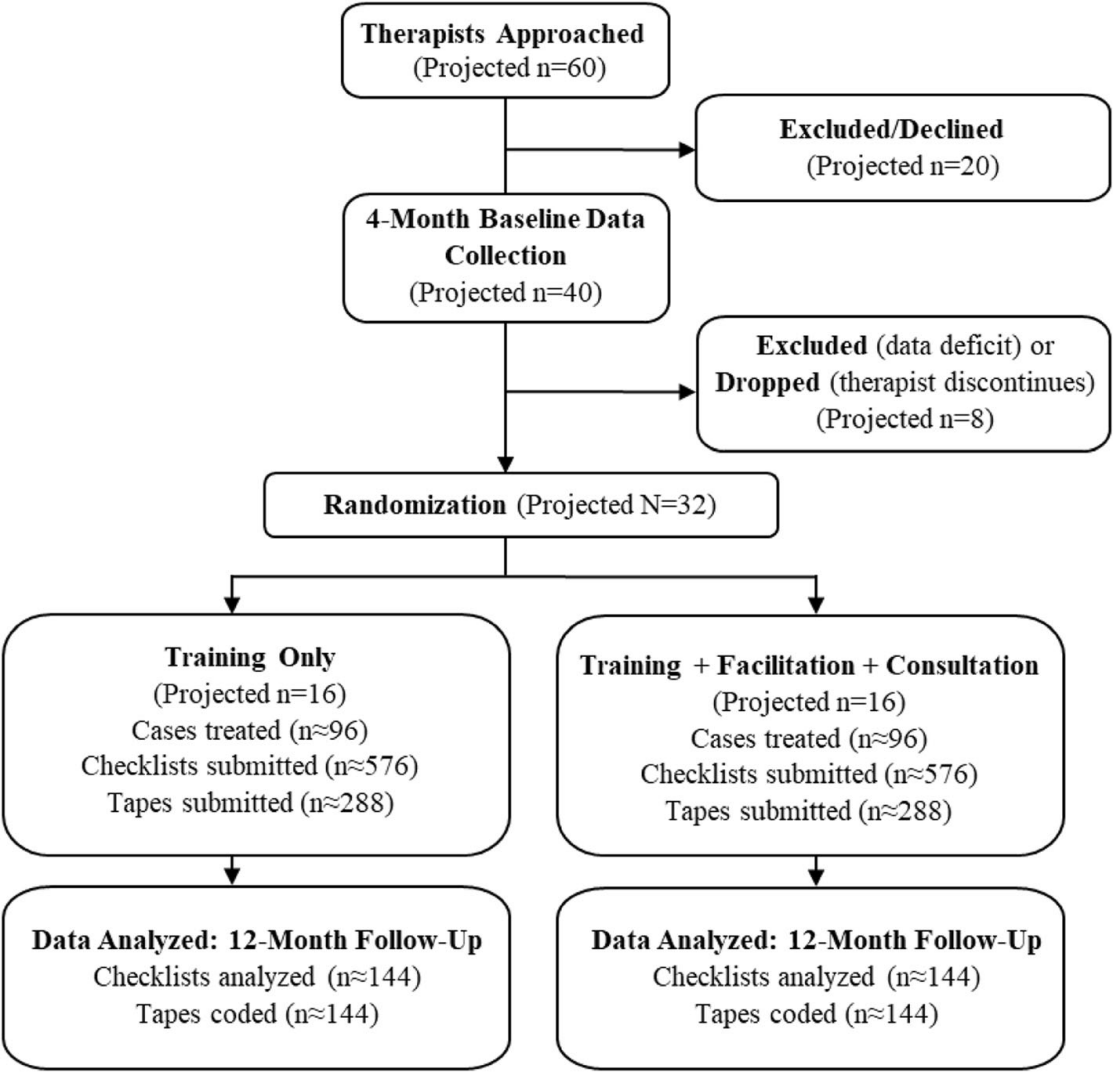
CONSORT flow chart of projected study enrollment

To host trial activities we will recruit four behavioral health outpatient clinics that attest to endorsing FT and CBT as staples of their clinical practice. All full-time clinicians will be eligible to participate. We project to enroll 32 therapists total (8 therapists per site [6 active slots plus 2 to replace dropouts]) treating 192 cases (24 therapist slots × 8 consenting cases/year). Treatment will average ~ 6 sessions/case (given routine therapy attrition), yielding ~ 1152 post-session therapist-reported checklists, about half with accompanying recordings (*n* ~ 576); half of these (~ 288 sessions) will be coded for EBT fidelity by research staff. Figure 3 shows a Consolidated Standards of Reporting Trials (CONSORT) flow chart of projected study enrollment. We will randomize volunteer therapists within each site to study condition at the start of the Implementation phase, given that MTFS-I procedures target individual therapists and their supervisors rather than entire agencies [24]. We will not randomize supervisors to condition, for two reasons. Because therapists in the Training Only condition will engage in solitary online training activities and will not review feedback reports or participate in expert consultation meetings with supervisors, we do not anticipate substantial supervisor “crossover” effects should a given supervisor oversee therapists in both conditions. Also, many clinics have only one supervisor, making it neither practical nor ecologically valid to randomize supervisors into condition. To guard against experimental contamination, supervisors will be asked not to review feedback reports in the presence of Training Only therapists.

Therapists enrolled in the study retain the right to withdraw consent at any time. The protocol will be discontinued at any site where procedures become burdensome or otherwise impinge on the routine performance of participating staff. Analysis of intervention impacts and potential harm will be continuous throughout the trial. In cooperation with the administration of partnering sites, investigators will provide full study debriefing and offer counseling referrals to any participant aggrieved or injured due to trial participation.

### Study measures

*The Inventory of Therapy Techniques for Adolescent Behavior Problems* (ITT-ABP) is a post-session therapist-reported fidelity tool that meets key criteria for pragmatic measures: relevance to stakeholders, low burden, broad applicability, strong psychometrics, and usefulness for data-driven decision-making (i.e., it is actionable). It requires 1–2 min to complete and was derived from validated observational fidelity tools for manualized treatments via a stakeholder-informed instrument adaptation process. The 13 FT and 15 CBT items each measure the extensiveness (i.e., thoroughness and frequency) with which each technique was used on a 5-point Likert-type scale: 0 = Not at all, 1 = A little bit, 2 = Moderately, 3 = Quite a bit, 4 = Extensively. Germane to rigor, ITT-ABP items derive from a validated observational EBT fidelity tool for manualized treatments that has shown strong construct and predictive validity in studies of treatment fidelity and fidelity-outcome links [14] with youth samples including conduct-disordered, depressed, and substance-using teens. The FT scale [28] and CBT scale (Hogue A, et al., Core elements of CBT for adolescent conduct and substance use problems: developmental psychopathology, clinical techniques, and case examples; submitted) were each enhanced via a comprehensive distillation process to identify the respective core treatment techniques of each approach as evidenced in controlled research with manualized treatment models for AEPs. Study therapists will complete the FT items, CBT items, or both, depending on which EBT(s) they elect to train. Baseline covariates will be assessed via three therapist-reported measures.

The *Organizational Social Context measure* [29] yields scaled scores that can be compared to national norms describing the organizational context of behavioral health clinics with regard to organizational culture, organizational climate, and work attitudes. The *Evidence-Based Practice Attitude Scale* [30] is a 15-item measure of clinician attitudes regarding appeal of EBTs, required use of EBTs, openness to trying EBTs, and unfavorable attitudes toward EBTs. The *Therapist Self-Reported EBT Proficiency* measure [31] averages therapists’ own judgments about their degree of allegiance to, and their perceived technical skill in, FT and CBT.

### Study interventions: MTFS-I components

MTFS-I intervention components are designed to be implemented flexibly in behavioral care settings, and therefore participating therapists are permitted to engage in concurrent training, supervision, and consultation.

#### MTFS-I training component

Online MTFS-I training will be used to increase validity in self-reporting on EBT fidelity and also to model high-fidelity EBT delivery. Online training has proven comparable or superior to in-person workshops in increasing clinical knowledge, self-reported use of treatment skills, and clinical proficiency [22, 32]. Front-line clinicians report comfort with online training, believe it to be efficacious [33], and believe it increases training accessibility and engagement [22]. MTFS-I training will be delivered weekly in two connected parts. (1) Self-monitored learning modules are brief descriptions and related clinical exemplars describing FT and CBT items from the ITT-ABP. Each learning module covers 2–3 items. Self-monitored training can reduce self-report biases of various kinds by providing continuous training in unbiased, accurate reporting [34]. (2) Mock session coding consists of 5–8 min video segments modeling examples of FT and CBT techniques on the ITT-ABP, illustrating a range from low to high extensiveness in order to support differentiated scoring. Trainees code segments directly after completing a corresponding self-monitored training module for those items in order to reinforce training elements. Trainees then submit ITT-ABP ratings for the given video segment and immediately view gold-standard scores, along with justification for the gold-standard scoring.

As discussed previously, these procedures mimic well-established observational training methods and leverage immediate corrective feedback on objectively rated samples of desired performance [25]. In addition to supporting self-report reliability and accuracy, these methods have strong potential for increasing EBT use via observational learning mechanisms prompted by modeling of quality EBT delivery. Although live coaching and guided skills practice are the most effective means to acquire new clinical skills [32], video-based modeling has also shown promise for increased EBT delivery (see, e.g., [35]).

#### MTFS-I feedback component

**Fig. 4:**
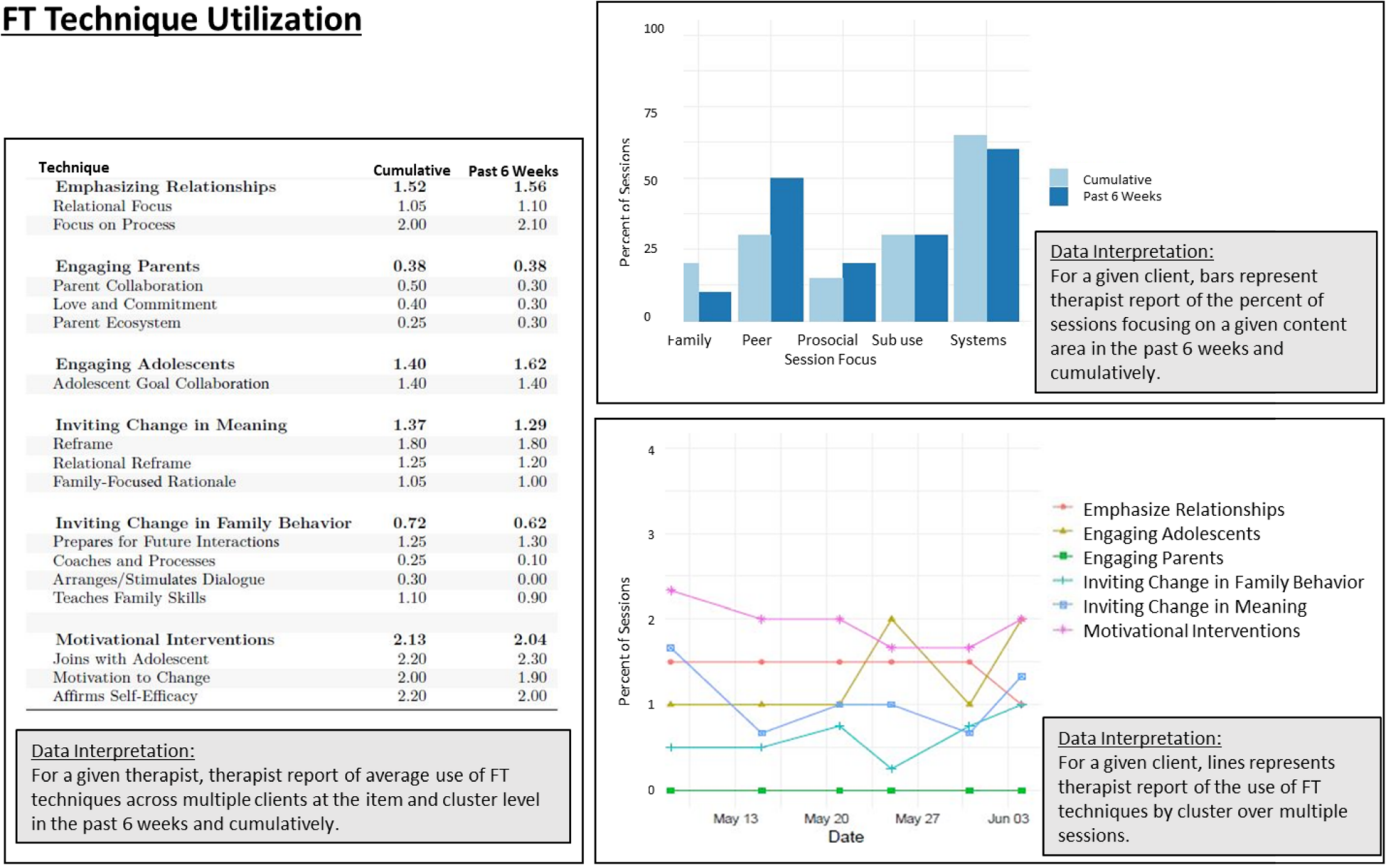
Sample of monthly feedback report based on ITT-ABP data

MTFS-I also features monthly feedback reports that summarize cumulative EBT use for each active case, based on therapist-reported ITT-ABP data. Feedback reports can contain (1) mean values for each EBT item and for the average EBT scale(s) (FT and/or CBT) aggregated at the client, therapist, and/or site levels; (2) aggregated EBT scale means plotted against benchmark fidelity levels. Figure 4 depicts content from a sample feedback report. A key predictor of adoption of innovative technology is fit between the technology and service context [36]. To promote compatibility and clinical relevance and increase collaborative investment in MTFS-I, after a lead-in period in which all sites gain familiarity with a basic report template, each site will define its own benchmark levels for EBT fidelity [23] to be specified in feedback reports, along with benchmarks drawn from research studies of FT and CBT models, respectively. Reports spur direct comparison between therapist self-reports of EBT delivery versus agency-specified benchmarks, precipitating data-driven self-correction that motivates movement toward fidelity goals. Data-based case feedback along multiple dimensions, including fidelity, is also thought to optimize change potential [23]. Each site will confer on the optimal design of feedback reports, user-friendly infographics of ITT-ABP data, the pragmatics of routine MTFS-I use, and potential organizational and staff-related facilitators and barriers to report use [37]. Feedback reports delivered to therapists will contain therapist-level data, whereas supervisor reports will contain agency-aggregated data, preserving therapist autonomy to share their own data in supervision.

### Study interventions: MTFS-I consultation

In keeping with evidence-informed guidelines for effective clinical facilitation (see [26]), MTFS-I consultation will focus on four interrelated strategies: discussion about recently viewed video vignette(s) (invoking retrieval practice and microlearning mechanisms), review of recently distributed feedback reports (cognitive rehearsal with increasing challenge), review of therapist-prepared case summaries combined with action planning for upcoming sessions (behavioral and cognitive rehearsal with variability), and live review of segments from submitted audiotapes (behavioral rehearsal with increasing challenge). Review of feedback reports will capitalize on data-driven decision-making, which has been shown to increase performance and productivity across a range of industry, education, and clinical service settings [38]. Sites will select the consultation format that fits best with extant site supervision practices: weekly 20–30 min by phone, bi-weekly 40–60 min by phone, or monthly 90–120 min in person. Although clinical consultation of this kind requires substantial resource commitments and extramural support, it is a common strategy shown to be feasible and valuable for scaling behavioral innovations across a variety of behavioral care systems, and community clinicians feel meaningfully supported by expert consultation [39]. Moreover, expert consultation on feedback reports has been shown to increase uptake of outcomes-focused MBC [13]. All study therapists will also receive routine technical assistance on MTFS-I system navigation and EBT data submission throughout the Implementation phase.

### Study procedures

All study data, including clinician demographics, self-reported fidelity checklists, and session audiotapes, will be obtained using data-secure procedures (e.g., ShareFile).

#### EBT fidelity and MTFS-I uptake data collection

ITT-ABP checklists and session audio recordings will be collected at all sites during the Baseline and Implementation phases. During the Implementation phase, therapists and supervisors will complete online MTFS-I training activities (self-monitored learning modules, mock vignette coding). To assess between-condition differences in consultation activities, therapists in both conditions will report on the extent to which training materials and feedback reports are discussed during clinical supervision.

#### Observational coding of EBT delivery

Session audio recording is a minimally intrusive procedure widely accepted by families and therapists in our previous studies that has proven feasible in usual care for youth behavioral health [19]. We will randomly select one recorded session from the Early phase (sessions 1–3) and the Later phase (sessions 4+) of treatment for each client to code with the observer version of the ITT-ABP [21]. Projecting 192 clients yielding 1.5 selected recordings apiece (factoring in treatment dropout after the Early phase sessions), we anticipate coding *n* ~ 288 recordings, of which 20% will be double-coded to establish observer reliability.

### Power analysis

Power to detect an experimental effect is based on *n* = 288 audiotaped sessions coded. The study is optimally powered (exceeding .80) to detect a between-condition effect size of *d* = .30 (small) or greater when data are combined across sites. Power calculations were conducted using Optimal Design 3.01 [40]. With nested designs, power is substantially affected by number of sites as well as clusters (therapists and clients) within site [41], and less so by cases (sessions) within cluster. Aggregating across site, and assuming 32 therapists (*unit of randomization*) treating 6 clients each and submitting 1.5 taped sessions for review (*unit of analysis*) and moderately sized within-cluster intraclass correlation coefficients (ICCs) (*ρ* = .05) [42], yields optimal levels of power (exceeding .80) for even small effects (*d* = .30) and increasing power (exceeding .90) with effect sizes of *d* = .40 and greater. Previous research has yielded effect sizes *d* = .40–.50 using a similar design [43].

### Data analysis plan

Study data will have a three-level nested structure: clients within therapists within sites (we will average across sessions for each client). The basic analytic approach for these nested data will be multilevel mixed effects models examining the effects of Phase (Baseline versus Implementation) and Condition (Training Only versus Training + Feedback + Consultation) on dependent variables aggregated across each time period: 4-month Baseline phase (prior to MTFS-I training) and 12-month Implementation phase. We will use maximum likelihood estimation for continuous variables and robust weighted least squares for categorical variables. We will model Site and Therapist as random effects in all models. We will include Therapist (age, sex, race/ethnicity, experience, EBT attitudes, EBT proficiency) and Client (age, sex, race/ethnicity) factors at their respective levels to examine potential effects. Effect sizes will be calculated using the standardized *d* effect size indicator, interpreted as the standardized difference between contrasts for Phase and Condition comparisons [41]. Aim 1 exploratory contrasts will examine the Phase (within-subjects) effect, contrasting Baseline versus Implementation across all sites in EBT self-monitoring (reliability, accuracy) and EBT technique use (averaging across FT and CBT techniques). Aim 2 will experimentally test the Condition (between-subjects) effect on MTFS-I uptake, EBT self-monitoring, and EBT technique use.

To analyze EBT self-monitoring, reliability between therapists and observers on FT and CBT technique use will be calculated using the ICC_2,2_ [44]. Conditions will be compared on the relative magnitude of ICCs for each EBT scale. To compare conditions on therapist accuracy, we will use statistical equivalence testing methods, applying the confidence interval approach [45] to examine whether therapist scores are equivalent to corresponding observer scores. To analyze EBT technique use, a series of multilevel mixed effects models will be conducted with a dummy-coded Condition variable included at the Therapist level to test the contrast of main interest; alpha will be adjusted to account for multiple contrasts. For the Aim 1 analyses of Phase contrasts, a product interaction terms will be included to examine Phase by Condition effects; for significant interactions, we will test simple effects by examining Phase contrasts separately within condition.

## Discussion

### Key study innovations

This study offers several innovations to the field of behavioral health services for AEPs. To our knowledge this is the first effort to adapt rigorous observational methods for training community clinicians to reliably and accurately self-monitor delivery of FT and CBT treatment techniques. A companion study by the investigative team [17] is developing an MTFS-I version focused on increasing family involvement in treatment and FT technique use among clinicians treating adolescents with substance use problems. The current study also leverages measurement feedback procedures to increase EBT utilization in everyday care. To our knowledge MTFS-I is the first feedback system in which providers can select their own EBT training preference (albeit from a limited set of two), delineate their own EBT fidelity benchmarks, and co-design their report templates so that feedback reports are tailored to local quality preferences. This provider-directed feature should strengthen therapist and agency investment in MTFS-I use and increases the system’s clinical relevance to therapists [23, 36], thereby reducing the provider resistance to adopting feedback systems that has plagued large-scale MBC implementation efforts [37].

By focusing on core practice elements of the FT and CBT approaches, MTFS-I diverges markedly from conventional efforts to disseminate EBTs via manualized treatment models relying on purveyor-driven quality procedures. The manual-driven strategy has encountered numerous barriers to implementing EBT models in routine care: high consultation costs, limited flexibility for selective treatment planning favored by clinicians, and sustainability limitations due to vicissitudes in local regulatory practices, purveyor commitment, and provider stamina to honor quality procedures [19]. The core elements approach is intended to mitigate many of these barriers and has accumulated an impressive research base in comparison to disorder-specific treatment manuals and usual care for youth behavior health problems [43]. MTFS-I procedures can flexibly fit within evolving accountability policies in behavioral healthcare [1] and could be generalized to virtually any set of core EBT techniques favored in youth or adult service settings. Also, MTFS-I procedures could be coupled with client outcomes tracking for an integrated feedback system.

### MTFS-I sustainability

Sustainability of innovations in behavioral treatment is an abiding concern of implementation science. MTFS-I procedures will be sustainable in behavioral care internationally only if (1) demands on provider time and resources are modest, and (2) providers independently value the benefits of MTFS-I and are motivated to use it. Regarding provider burden, MTFS-I is anchored by user-centered design features that minimize staff time commitments. The time investment is about 20 min per week (per EBT approach) for online training and ITT-ABP data submission, plus review of monthly feedback reports. This commitment appears feasible given the expected benefits of increased EBT fidelity and the caseload-specific relevance of the training and feedback components. The flexibility of MTFS-I components also promotes their acceptability: Feedback report templates can be tailored to the specifications of therapists, supervisors, administrators, and/or regulatory agencies; and clinical supervisors have appreciable latitude in how to incorporate feedback report data into supervision sessions. The online MTFS-I platform is highly conducive to adaptation over time as procedures become routinized within a given agency. The time and resource commitment for ongoing MTFS-I technical assistance and, as necessary, expert consultation is de rigueur for clinical training experiences [26] and may be cost-efficient if these procedures prove to significantly boost MTFS-I system uptake and fidelity effects.

Regarding provider motivation, there are several direct benefits and strong incentives for agencies to sustain MTFS-I. Our own research on EBTs in routine care in the USA (e.g., [21, 31]) has shown that clinicians are motivated to submit self-monitoring fidelity data and engage in discussions related to quality improvement if they believe these activities enhance their clinical knowledge and skillsets and are valued by supervisors and administrators. Also, EBT implementation procedures that are grounded in pragmatic quality metrics such as the ITT-ABP will likely increase in value to agencies as accountability contracting (e.g., value-based purchasing) becomes commonplace. Finally, MTFS-I procedures bypass two major obstacles to implementing MFS technology in behavioral care [37] by (1) providing ongoing and accessible training experiences to all system users (including supervisors and program administrators) and (2) ensuring that feedback data are systematically incorporated into everyday workflow and supervision.

### Trial status

This clinical trial (registration ClinicalTrials.gov NCT03722654; original protocol, October 29, 2018) has not yet enrolled participants. We anticipate enrolling initial participants in November 2019 and completing recruitment in November 2020.

## Supplementary information

**Additional file 1.** Study committees/teams.

**Additional file 2.** Informed consent form.

**Additional file 3.** SPIRIT 2013 checklist: recommended items to address in a clinical trial protocol and related documents.

## Data Availability

The original study protocol is publicly available on ClinicalTrials.gov, registration number NCT03722654. The datasets generated and analyzed during the current study are available from the corresponding author on reasonable request.

## Abbreviations

AEP: Adolescent externalizing problem
CBT: Cognitive behavioral therapy
EBT: Evidence-based treatment
FT: Family therapy
ITT-ABP: Inventory of Therapy Techniques for Adolescent Behavior Problems
MBC: Measurement-based care
MFS: Measurement feedback system
MTFS-I: Measurement Training and Feedback System for Implementation
