# Supplementary material for: Measurement training and feedback system for implementation of evidence-based treatment for adolescent externalizing problems: protocol for a randomized trial of pragmatic clinician training
Source: Trials. 2019 Dec 10;20:700. doi: 10.1186/s13063-019-3783-8 (PMC6905067; doi:10.1186/s13063-019-3783-8)
Supplement: Supplementary file 1 — Additional file 1. Study committees/teams. [file 13063_2019_3783_MOESM1_ESM.docx]

Additional file 1

**Study Committees/Teams**

**Principal Investigator**

Design and conduct of MTFS-I

Preparation of protocol and revisions

Publication of study reports

**Steering Committee**

(see title page for members)

Agreement of final protocol

All lead investigators will be steering committee members.

Recruitment of clinics with the PI

Reviewing progress of study and if necessary agreeing changes to the protocol to facilitate the smooth running of the study.

**Data Manager Study Coordinator**

Maintenance of trial IT system and data entry

Data verification

**Local Champions**

In each participating clinic a liaison (program director/clinical supervisor) will be identified, to be responsible for coordinating training workshops and maintaining open communication with the study team regarding data submission, technical assistance needs, and staff turnover and feedback.

**Data Safety and Monitoring Board**

An independent group of experts charged with reviewing study data for quality and integrity, adherence to the protocol, participant safety, study conduct and progress, and making determinations regarding study continuation, modifications, and suspensions/terminations will be appointed. The DSMB will work in conjunction with the PI and IRB.
